# Home-based rehabilitation using a soft robotic hand glove device leads to improvement in hand function in people with chronic spinal cord injury:a pilot study

**DOI:** 10.1186/s12984-020-00660-y

**Published:** 2020-03-05

**Authors:** Bethel A.C. Osuagwu, Sarah Timms, Ruth Peachment, Sarah Dowie, Helen Thrussell, Susan Cross, Rebecca Shirley, Antonio Segura-Fragoso, Julian Taylor

**Affiliations:** 1 National Spinal Injuries Centre, Stoke Mandeville Hospital, Mandeville Road, Aylesbury, HP21 8AL UK; 2 Bucks Healthcare Plastics, Stoke Mandeville Hospital, Mandeville Road, Aylesbury, HP21 8AL UK; 3 Instituto de Ciencias de la Salud, Talavera de la Reina, Castilla-La Mancha, 45600 Spain; 4 Sensorimotor Function Group, Hospital Nacional de Parapléjicos, SESCAM, Toledo, 45071 Spain; 5 Harris Manchester College, University of Oxford, Oxford, OX1 3TD UK

**Keywords:** Activity-based neurorehabilitation, Home-based, Self-administered therapy, Neurorehabilitation, Grasp strength, Activities of daily living

## Abstract

**Background:**

Loss of hand function following high level spinal cord injury (SCI) is perceived as a high priority area for rehabilitation. Following discharge, it is often impractical for the specialist care centre to provide ongoing therapy for people living with chronic SCI at home, which can lead to further deterioration of hand function and a direct impact on an individual’s capability to perform essential activities of daily living (ADL).

**Objective:**

This pilot study investigated the therapeutic effect of a self-administered home-based hand rehabilitation programme for people with cervical SCI using the soft extra muscle (SEM) Glove by Bioservo Technologies AB.

**Methods:**

Fifteen participants with chronic cervical motor incomplete (AIS C and D) SCI were recruited and provided with the glove device to use at home to complete a set task and perform their usual ADL for a minimum of 4 h a day for 12 weeks. Assessment was made at Week 0 (Initial), 6, 12 and 18 (6-week follow-up). The primary outcome measure was the Toronto Rehabilitation Institute hand function test (TRI-HFT), with secondary outcome measures including pinch dynamometry and the modified Ashworth scale.

**Results:**

The TRI-HFT demonstrated improvement in hand function at Week 6 of the therapy including improvement in object manipulation (58.3 ±3.2 to 66.9 ±1.8, p ≈ 0.01), and palmar grasp assessed as the length of the wooden bar that can be held using a pronated palmar grip (29.1 ±6.0 cm to 45.8 ±6.8 cm, *p* <0.01). A significant improvement in pinch strength, with reduced thumb muscle hypertonia was also detected. Improvements in function were present during the Week 12 assessment and also during the follow-up.

**Conclusions:**

Self-administered rehabilitation using the SEM Glove is effective for improving and retaining gross and fine hand motor function for people living with chronic spinal cord injury at home. Retention of improved hand function suggests that an intensive activity-based rehabilitation programme in specific individuals is sufficient to improve long-term neuromuscular activity. Future studies should characterise the neuromuscular mechanism of action and the minimal rehabilitation programme necessary with the assistive device to improve ADL tasks following chronic cervical SCI.

**Trial registration number:**

Trial registration: ISRCTN, ISRCTN98677526, Registered 01/June/2017 - Retrospectively registered, http://www.isrctn.com/ISRCTN98677526

## Introduction

High level spinal cord injury (SCI) often involves impairment of both upper and lower limb function, with the deficit in hand function perceived by affected individuals as a high priority problem [1, 2]. Activities of daily living (ADL) such as feeding, grooming, and dressing become difficult without normal hand function, and often oblige the individual to be reliant on caregiver assistance even for the most basic tasks. Furthermore, sensorimotor fine hand function essential for object manipulation is severely impaired in people with tetraplegia.

Despite the intensive hand rehabilitation therapy or compensatory strategies provided by the spinal cord injury centre during the sub-acute rehabilitation phase, significant impaired function often continues to be present during chronic SCI. This presents a challenge in how an effective self-administered home-based therapy programme could be provided.

Several assistive and rehabilitative devices have been designed to assist with ADL in the home in people with chronic SCI [3]. However, many of these devices are complex and comprise large exoskeletons [4–10] or soft bulky materials mounted on the back of the hand [11, 12] that could make object manipulation difficult. The success of self-administered hand therapy at home and in the community will depend on the use of simple orthotic and rehabilitative devices that are additionally portable, cost-effective, user-friendly and importantly easy to don and doff. Current research in the area of soft robotics has led to development of deformable hand glove devices without restrictive exoskeletons [13–16]. Soft robotic gloves are promising devices for self-administered hand therapy at home and in the community.

Several studies have investigated self-directed [17], supervised [18] or self-administered [19–22] upper limb therapy on people with stroke [23–25]. The studies investigated several techniques including the use of novel robotic systems [26–28] to deliver rehabilitation therapies at home [29–34]. The results from most of these studies showed that stroke participants improved in several outcomes including ADL performance, which were maintained for several weeks. A literature search demonstrated that, when compared to stroke, only a limited number of unsupervised home-based upper limb rehabilitation studies for participants with high level SCI are available. One home-based rehabilitation programme in high level SCI compared a conventional therapy with Rejoyce Excercise therapy which involved functional electrical stimulation in addition to computer games and showed that participants gained significant improvement in hand function compared with conventional therapy over a minimum of 16 weeks [35]. The study showed that home-based therapy previously applied to stroke patients can similarly be beneficial for people living with high level SCI.

The soft extra muscle (SEM) Glove by Bioservo Technologies AB, Sweden, is a device that could have a therapeutic effect in people with impaired hand function after high level SCI. The compact light-weight device is designed to partly support grasp function by providing additional finger flexion strength [36, 37]. It is a servo device which uses artificial tendons attached only alongside the thumb, middle finger and ring finger. The tendons are connected to electrical motors which actuate thumb and finger movements by creating pulling forces. The control algorithm is based on signals from tactile sensors located on the tip of the thumb, middle finger, ring finger and on the palm [36]. The device detects intention to grip or manipulate an object via the tactile sensors and applies proportional finger flexion strength to facilitate strong grip. The orthotic effect of the device has previously been tested in a study, among individuals with impaired hand function due to aging [37]. The participants in that study accepted the device and their study task performance improved with repetitions. The SEM Glove is designed to encourage active engagement in a motor task. This is essential to achieve improved voluntary motor control mediated by the motor cortex [38, 39], and also provides feedback to the sensorimotor system [38, 40–43]. Its use may therefore potentially lead to motor learning due to neuroplasticity [40, 43].

The objective of this study was to assess whether self-administered home-based hand therapy using the SEM Glove system may lead to improvement in hand function, leading to improved ability to perform ADL.

## Methods

### Data collection

#### Participants

Fifteen people (mean age 50.3, range 33 - 60) diagnosed with SCI were recruited in this study. This sample size was chosen to facilitate determination of the effectiveness of the intervention. Inclusion criteria were: age between 18 - 65 years old, with a chronic (>12 months post injury) motor incomplete (American spinal injury association, ASIA, impairment scale, AIS grade of C or D) tetraplegia with a neurological level between C2 - C8. Exclusion criteria included a known neurological condition or comorbidity (e.g. brain injury) and an inability to understand verbal or written instructions in English. Informed consent was obtained from all participants according to the approved protocol (UK Health Research Authority and London - City and East REC). Demographic data of the participants is shown in Table 1.

**Table 1:** Demographic and clinical SCI characteristics data of the recruited study participants

Parti #.	Age	Gender	N. level	AIS	UEMS	Time since SCI(m)	T. Hand	# A. Sessions
CP01	45	F	C2	D	20	17	R	4
CP02	44	M	C3	D	25	14	R	4
CP03	33	F	C2	C	13	54	L	1
CP04	54	F	C5	D	9	120	R	1
CP05	55	M	C2	D	18	27	L	4
CP06	51	F	C2	C	14	192	L	4
CP07	51	M	C4	D	24	28	R	1
CP08	50	M	C2	C	19	25	L	4
CP09	59	M	C5	D	16	14	L	4
CP10	56	M	C3	D	21	20	R	2
CP11	38	M	C4	D	13	39	R	4
CP12 ^*a*^	56	M	C3	UT	22	68	L	4*
CP13	57	M	C4	D	23	60	R	2
CP14	60	M	C3	D	25	16	R	4
CP15	46	M	C3	D	12	63	L	4

Parti. #, Participant number; N. level, Neurological level; AIS, American Spinal Injury Association Impairment Scale; UEMS, Upper Extremity Motor Score; m, months; T. hand, Treated hand; # A. Sessions, Number of assessment sessions; F, Female; M, Male; R, Right; L, Left; UT, Untestable. ^*a*,∗^ participant had four sessions but only two were applicable to the study analysis

#### Setting

This study was set at the National Spinal Injuries Centre (NSIC), Stoke Mandeville Hospital, Buckinghamshire Healthcare NHS Trust, Aylesbury, HP21 8AL.

#### Study design

The study follows the interventional longitudinal clinical trial design. Each Participant was scheduled to spend 18 weeks in the trial. Intervention was given to the participants in the first 12 weeks of their participation. They were assessed at various time points including a follow up after the intervention period. The study was not controlled because firstly and more importantly, the participants are at the chronic stage and are therefore not expected to spontaneously recover, and secondly, there were a limited number of potential participants.

#### Intervention

Participants were required to don the SEM Glove at home for a minimum of 4 h a day (straight or in short sessions) for a total period of 12 weeks. They were asked to maintain a basic diary to ensure protocol adherence and to record ADL completed while wearing the glove. Participants were asked to perform a study defined set of tasks in which they used the glove each day to grasp and release a softball 30 times repeatedly and to perform 30 trials of simulated drinking from a drink can, including picking the can up from a table and replacing it. They were also required to eat a meal with a fork or spoon and write their name and address. The study defined set of tasks were used to set a minimum standard usage and to motivate the participants. Following these set tasks the participants were instructed to continue using the glove to fit with their daily activities. The study team contacted the participants by telephone approximately once a fortnight to check how they were progressing and if they had any issue to report. The study did not involve any visit to the participants’ homes by the study team.

#### Assessments

Participants were scheduled to attend four assessment sessions named Initial, Week 6, Week 12 and Week 18. During these sessions, participants were assessed using various scales. The assessment tools included the Toronto Rehabilitation Institute hand function test (TRI-HFT), pinchmeter for grip strength, modified Ashworth scale (MAS), Quebec user evaluation of satisfaction with assistive technology (QUEST version 2.0 [44]) and short-form 36 (SF-36) for health state. Standard procedures were followed in all assessments and no extra upper limb support was provided.

The Toronto Rehabilitation Institute hand function test (TRI-HFT) [45] was used to measure gross motor function of the hand in all sessions of the study. It comprises five components: object manipulation, rectangular wooden blocks, instrumented cylinder, instrumented credit card and wooden bar. Object manipulation and rectangular wooden blocks components, scored using a unitless numeric scale with a range between 1 and 7, involved assessing the capacity to grossly manipulate many objects e.g. paper sheet, dice, credit card, book, phone, pencil, etc. encountered in ADL. The wooden bar component relates to estimation of moment of a load (recorded in this study as length) that can be sustained with a palmar grasp. The instrumented cylinder and instrumented credit card components of the TRI-HFT involve assessing the torque (presented here as force) of palmar grasp and resistive force of lateral pinch respectively. Parts of this test were video recorded.

Due to the nature of data acquisition for the instrumented cylinder and credit card components of the TRI-HFT which involves pulling on a breakable string attached to a dynamometer, force was limited to 30 N for reliability and safety of the assessor and participant. For this reason the tests were stopped if a participant reached 30 N.

A pinchmeter was used to assess the participants for strength of three grip types: jaw, key and tip to tip, using the Biometrics Ltd. P200 pinchmeter. For each grip type, participants were asked to squeeze the pinchmeter at maximum voluntary force before releasing it; this was repeated three times. Raw strength data were recorded using Biometrics DataLINK data acquisition software. This assessment was performed during all sessions of the study.

The Modified Ashworth scale (MAS) [46] was used to rate muscle tone/stiffness during passive movement of the upper limb including abductors, adductors, extensors and flexors of the shoulder, extensors and flexors of the elbow, wrist, fingers and thumb. MAS scores were calculated as the sum of muscle tone rated during extension and flexion of the joints. The scale ranges from ‘0 = normal’, ‘1’, ‘1+’, ‘2’, ‘3’, and ‘4= worst’. Participants were assessed in all sessions using this 6-point ordinal scale for the hand treated in the study.

#### Procedure

**Fig. 1:**
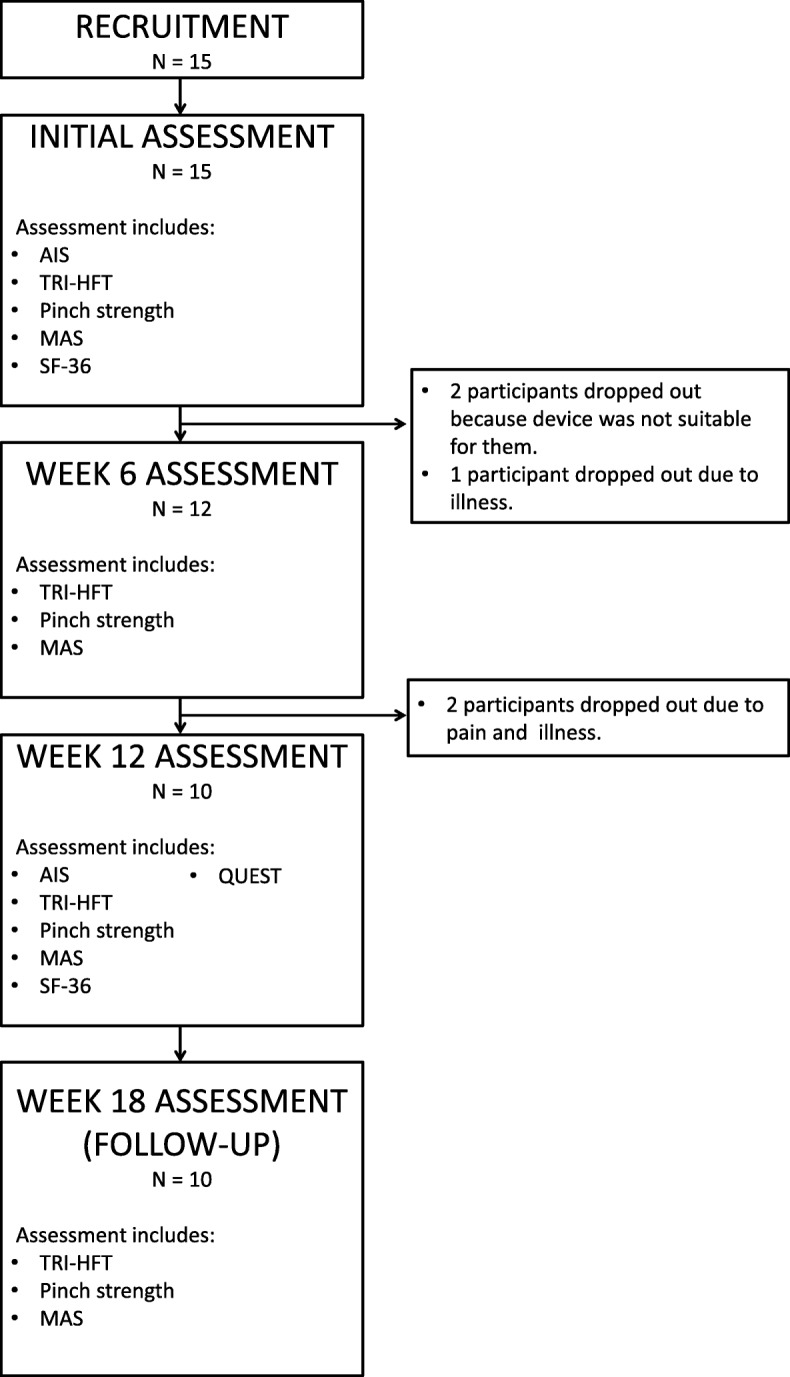
Study flow diagram. Study flow diagram showing number of participants and the assessments performed in each session. American spinal injury association impairment scale, (AIS); TRI-HFT, Toronto Rehabilitation Institute hand function test; Pinch strength, Pinchmeter for grip strength; MAS, Modified Ashworth scale; QUEST, Quebec user evaluation of satisfaction with assistive technology; SF-36, Short-form 36

Following recruitment participants were invited to an *Initial* session where they were administered with the assessments including TRI-HFT, pinchmeter dynamometry, MAS and SF-36, without the glove. This session also involved ASIA examination to determine their exact SCI classification. During this session they selected with a therapist which hand is most suitable, were fitted with and given the SEM Glove device to use in their own homes to accomplish the study defined tasks and their usual ADL. The device was set such that the participants were only required to push a button to power it (ON and OFF). If they wanted, there were extra buttons they could push to choose different grip strengths from the glove that were pre-programmed for them by an occupational therapist. After using the device at home for a period of six weeks, they were invited back for *Week 6* for a minor assessment session including TRI-HFT, pinchmeter dynamometry and MAS. Following this session participants continued to use the device at home for a further six weeks after which they were invited back for *Week 12*, an assessment session similar to the Initial. The session additionally included assessment with the QUEST which allowed the participants to evaluate the device and related services. In this session participants returned the glove. A further six weeks after returning the glove they were invited for *Week 18*, a follow up assessment session similar to Week 6. The study flow diagram is shown in Fig. 1.

### Analysis

In addition to the analysis with all four sessions together, a separate analysis was performed when applicable to compare Initial with Week 6 sessions in order to include participants who did not complete all the sessions. Data from participants who did not complete up to two sessions were discarded.

#### Toronto rehabilitation institute hand function test

Ceiling effects were observed in each component of TRI-HFT. Data for a subject for a component was discarded if the ceiling effect (when the test score equals the upper limit of scale) was observed in all or the first two sessions [47]. The *part* of instrumented cylinder and credit card components (unrelated to the main force measurement) that assessed whether a participant could hold the instrumented items was not used in this study.

#### Grip strength using pinchmeter

For each grip type, the trial with the highest value was selected as the grip strength since this represents maximal strength. The resulting grip strength across assessment sections was subjected to a t-test and repeated measures analysis of variance. The averages across participants are presented.

#### Modified ashworth scale

Due to the ordinal nature of MAS scale which makes it ambiguous to interpret sum of scores, statistical tests were performed for session effect separately for each upper limb movement assessed. However an aggregated score was obtained for each level of the scale as its frequency of occurrence in each assessment session. Participants who achieved the maximum score (MAS score = 0) on the MAS test in all sessions (i.e ceiling effect) for each upper limb movement tested were not used in further analysis. In order to statistically test for session effect, the raw MAS scores were transformed to 6-point ordinal numbers, ‘0’ to ‘5’.

#### Short form 36

Short form 36 (SF-36 [48]) was analysed by calculating the Standard Gamble health state valuation using an algorithm kindly donated by Prof. John Brazier, University of Sheffield [49].

### Statistics

Analyses were performed under MATLAB R2014a and Microsoft Excel using the statistics toolbox and Data Analysis Toolpak respectively. When CP12 and participants who did not complete all sessions were included in the analysis, student’s t-test and Wilcoxon signed rank test were appropriately used to compare results between the Initial and Week 6 sessions. One-way repeated measures and Friedman ANOVA tests were conducted to assess session effect between the Initial and Week 6, Week 12, and, Week 18. ANOVA tests were followed with three pairwise comparisons between the Initial and each of the other sessions.

Statistical significance level was set at 0.05. For the parametric and non-parametric (Friedman test) ANOVA, the significance level was adjusted to 0.0167 using the Bonferroni correction for multiple comparison. The unadjusted *p*-values are presented and unless otherwise stated mean scores are reported together with standard error (i.e mean ± std error).

## Results

### Participants’ experience

Two participants withdrew after the Initial assessment session citing that the glove was not as helpful as expected, ‘got in the way’ during ADL, and did not fit comfortably due to a part placed on the palmar side of the device. One of the participants also thought that the device-induced movement was opposing that of a surgically operated thumb. Another participant could not continue after the first session due to illness. This participant did not use the device at all. Two further participants withdrew after the Week 6 assessment session citing pain which they believed was related to glove use and unrelated illness. Participant CP12 completed all assessment sessions but did not use the device during the first six weeks due to misplacement.

There was no major adverse event reported in this study. One of the participants who withdrew after the second assessment session cited pain related to the use of the glove as mentioned earlier. One other participant who completed the study also wrote in the feedback and spoke of pain on the involved limb after long period using the glove.

Most participants were able to don the glove without help during the Initial assessment session. Only participants in this study with more severe hand impairment required a carer’s assistance to use the glove. According to the completed diary, participants used the glove for tasks such as eating a burger, holding handrail while climbing the stairs, vacuuming, dusting, grooming, building models, food preparation and other ADL. Table 2 shows the usage time for participant who completed at least the Week 6 assessment and returned their diaries. Most participants reported in the diary that they used the glove 0.3–6 h daily apart from, according to some, when on holidays or during ill health. But one participant reported taking the glove along on holidays. The mean time participants reported that they spent using the glove was 119.8 ±30.7 h (*n* = 9) (mean ± std error) between the Initial and Week 6 assessment and 91.4 ±30.9 h (*n* = 6) between Week 6 and Week 12. These mean usage times are not directly comparable because as shown in Table 2 three participants who returned their diaries during the Week 6 assessment session did not do so during the Week 12 assessment session. Because the glove can be donned and used only when required in ADL, this dosage is only an estimation (particularly an over estimation) of an actual total use in a continuous task. For these reasons it is risky to speculate on the difference between these numbers. However, it can be speculated that participants were more excited about the device at the beginning and were likely to plan to start the study when they were in good health and had fewer commitments (e.g holidays). They therefore had more interest and time to commit at the early stage of the study than later.

**Table 2:** Estimated total SEM Glove usage time from participants’ diary

Participant ID	Week 1 - 6 (h)	Week 6 - 12 (h)
CP01	117.5	60.5
CP05	143.9	na
CP06	130.8	82.0
CP08	37.0	17.0
CP09	56.0	22.0
CP10	52.8	na
CP13	51.8	na
CP14	153.6	169.1
CP15	334.7	197.7
**Mean**	**119.8 ±30.7**	**91.4 ±30.9**

Data is only available for participants who finished Week 6 assessment. Some data are not available because some participants did not return their diary. h, Hour; na, Not available

From verbal feedback participants generally found the glove useful although they would prefer it if the device was water resistant, washable by full water immersion, covered all digits and even more compact and comfortable for longer daily use. They reported that it was not practical to don and doff the device between wet activities such as food preparation. Some participants noted that they needed a more secure Velcro strap to hold the device in position along the forearm because the current strap kept falling off. One participant said that the control unit housing the motors was heavy enough to pull down his trousers when the unit was clipped on, and asked if the device could become even lighter. However, the feedback and the diary suggested that participants generally accepted and actively used the device while performing various ADL and would like it to be developed further for people with spinal cord injury.

The user acceptance of the device was evaluated using the QUEST questionnaire. The mean rating for the ‘Assistive device’ subscale was 3.8 ±0.2 and 4.7 ±0.1 for the ‘Services’ sub-scale, with both scores equivalent to ‘more or less satisfied’ and ‘quite satisfied’, respectively. The total QUEST score was 4.1 ±0.2 out of 5.0 which is equivalent to ‘quite satisfied’. The participants most frequently selected Effectiveness, Comfort, and Ease to use, as the most important three items ranked in order.

A maximum of five participants could be recruited in this study at a time because there were five glove devices available. One device was often left as a spare. At the end of a participation, a device was transferred to another participant. Although the devices were reused by the participants, there were only two instances of device malfunction during the study. The participants involved reported that the malfunction occurred during normal use of the device. In these cases participants were supplied with a replacement and were able to continue their participation.

### ISNCSCI sensorimotor scores

The international standards for neurological classification of spinal cord injury (ISNCSCI) scores were computed from the ASIA assessment and compared between the Initial and Week 12 assessment sessions for 9 individuals for whom there is complete data. The data included the UEMS for the tested hand and the mean sensory score for the ipsilateral hand between the C5 and T1 dermatomes. No change in UEMS was identified (18.7 ±1.6 vs. 18.6 ±1.7, *p* = 0.93). No change was detected either for the total Light Touch (LT) score (35.1 ±2.2 vs. 36.3 ±3.4, *p* = 0.33) or mean LT dermatomal score (1.4 ±0.1 vs. 1.4 ±0.1, *p*= 0.56). In contrast the pinprick (PP) score indicated an increase towards normal at Week 12 (23.4 ±5.6 vs. 28.3 ±5.3, *p* = 0.08). A significant increase in PP score to a normal level was identified however when the mean PP sensory score was calculated for the ipsilateral C5-T1 dermatomes (1.0 ±0.2 vs. 1.5 ±0.1, *p* = 0.02).

### Toronto rehabilitation institute hand function test

**Fig. 2:**
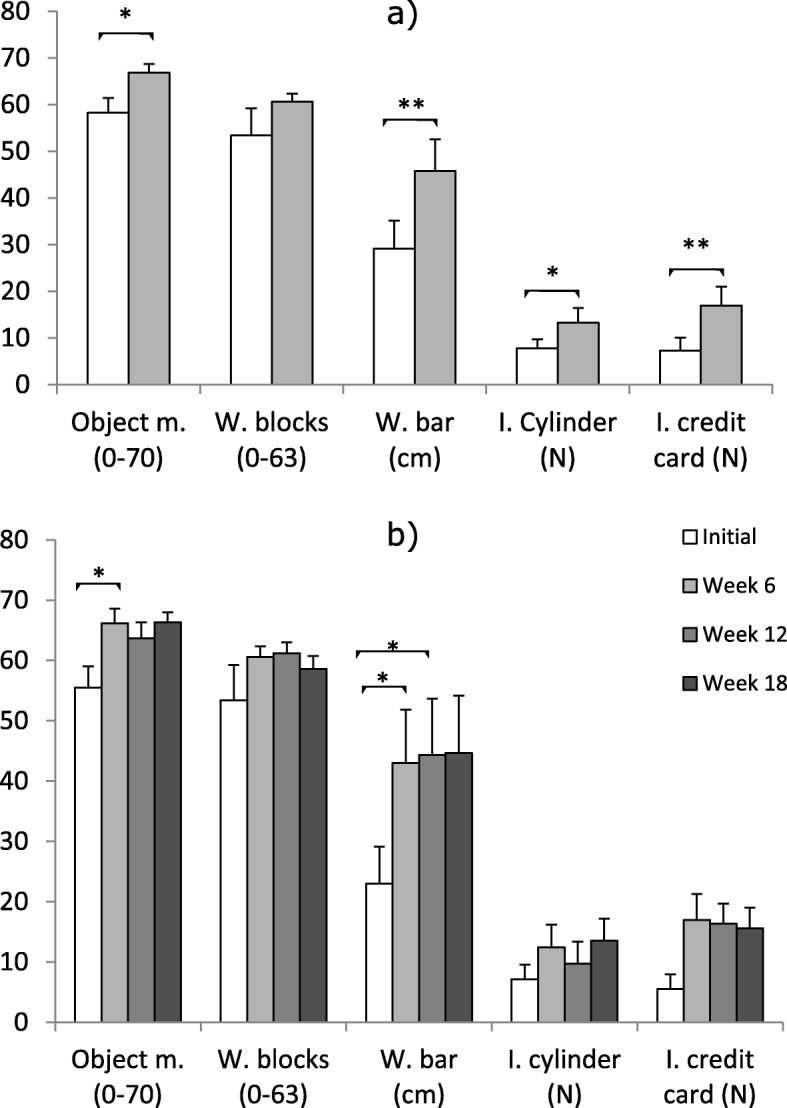
Results of TRI-HFT. Mean TRI-HFT scores for participants who completed **a** Week 6 and **b** Week 18. The brackets in the group names of the bars indicate the scales. In **a** Object m., *n*=8; W. Blocks, *n*=5; W. bar, *n*=8; I Cylinder, *n*=11 and I. credit card, *n*=8. In **b** Object m., *n*=6; W. Blocks, *n*=5; W. bar, *n*=6; I Cylinder, *n*=6 and I. credit card, *n*=6. Object m., Object manipulation; W. Blocks, Wooden blocks; W. bar, Wooden bar; I, instrumented. In **a** *, *p* <0.05; **, *p* <0.01; in **b** *, *p* <0.016


The plot for all the components of TRI-HFT is shown in Fig. 2. Results of the separate analysis to compare the Initial and Week 6 sessions showed that in all but the wooden block component of the TRI-HFT participants were significantly better in Week 6 compared to the Initial assessment session. For the object manipulation component, mean score significantly improved from 58.3 ±3.2 in the Initial to 66.9 ±1.8 during the Week 6 assessment session (*n* = 8, *p* = 0.0109). There was no statistical significant difference (*n* = 5, *p* = 0.313) between the mean scores for the wooden blocks component which was 53.4 ±5.8 during the Initial and 60.6 ±1.7 during the Week 6 assessment session. For the instrumented cylinder component, the mean significantly improved from 7.7 ±1.9 N during the Initial to 13.3 ±3.1 N during the Week 6 assessment session (*n*=11, *p* = 0.0133). While for the instrumented credit card component, the mean force significantly improved from 7.3 ±2.8 N during the Initial to 17.0 ±4.0 N during the Week 6 assessment session (*n*=8, *p* = 0.00934). And for the wooden bar component, mean score significantly improved from 29.1 ±6.0 cm during the Initial to 45.8 ±6.8 cm during the Week 6 assessment session (*n* = 8, *p*= 0.00542). These results summarised in Fig. 2a suggest improvement in hand function of the participants after using the glove. Anonymised video recording showing functional improvement during the TRI-HFT assessment is provided in Additional files 1, 2 and 3.

Additional file 1: A video footage of a participant completing a task involving manipulation of a can from Toronto Rehabilitation Institute hand function test. The participant was instructed to lift a can off the surface and manipulate it including simulation of drinking and turning the palm up and down while still holding it. The video shows the performance on this task for the participant during the baseline assessment (Initial), after six weeks of using the device (Week 6), after 12 weeks of using the device (Week 12) and during the follow up assessment session (Week 18). The task was more difficult and was performed less smoothly during the Initial assessment compared with the other assessment sessions.


The mean scores for participants who completed all sessions are shown in Fig. 2b. For the object manipulation component, Mauchly’s test indicated that the assumption of sphericity had been violated, *χ*^2^(5)=13.307, *p* = 0.0207 (*n*=6). Greenhouse-Geiser correction was used to estimate sphericity (*ε*= 0.388). Following the correction for assumption of sphericity, the repeated measures analysis for the object manipulation component scores revealed no main effect of assessment session F(3,15)= 5.321, *p*= 0.058944. However post hoc analysis using Bonferroni correction for multiple comparison revealed a significant increase in hand function from the Initial assessment session (55.5 ±3.5) to Week 6 (66.2 ±2.4, *p* = 0.013358) but not for Week 12 (63.7 ±2.7 N, *p* = 0.14666), and Week 18 (66.3 ±1.6 N, *p* = 0.051762).

The repeated measures ANOVA for the wooden blocks component scores did not reveal main effect of assessment session, F(3,12)=1.2988, *p*=0.31988 (*n*=5). For the instrumented cylinder component the test revealed a main effect of assessment session, F(3,21)=3.7253, *p*= 0.027208 (*n*=8). Post hoc analysis using Bonferoni correction for multiple comparison did not reveal significant difference between the Initial assessment session (7.1 ±2.4 N) and Week 6 (12.4 ±3.8 N, *p* = 0.059285), and Week 12 (9.8 ±3.6 N, *p* = 0.29287), and Week 18 (13.6 ±3.6 N, *p* = 0.05098). Similarly for the instrumented credit card component the repeated measures ANOVA revealed main effect of assessment session, F(3,15)=4.3452, *p* = 0.021595 (*n*=6). Again post hoc analysis using Bonferoni correction for multiple comparison did not reveal significant difference between the Initial assessment session (5.5 ±2.4 N) and Week 6 (16.9 ±4.4 N, *p* = 0.018102), and Week 12 (16.3 ±3.3 N, *p* = 0.032797), and Week 18 (15.6 ±3.4 N, *p* = 0.051542).

For the wooden bar component scores, the ANOVA revealed a main effect of assessment session, F(3,15)= 11.973, *p*= 0.0002914 (*n*=6). Post hoc analysis using Bonferoni correction for multiple comparison revealed a significant increase in hand function from the Initial assessment session (23.0 ±6.1 cm) to Week 6 (43.0 ±8.8 cm, *p* = 0.0086347), Week 12 (44.3 ±9.3 cm, *p* = 0.0076498), with an almost significant change for Week 18 (44.7 ±9.5 cm, *p* = 0.017671).

### Grip strength using pinchmeter

**Fig. 3:**
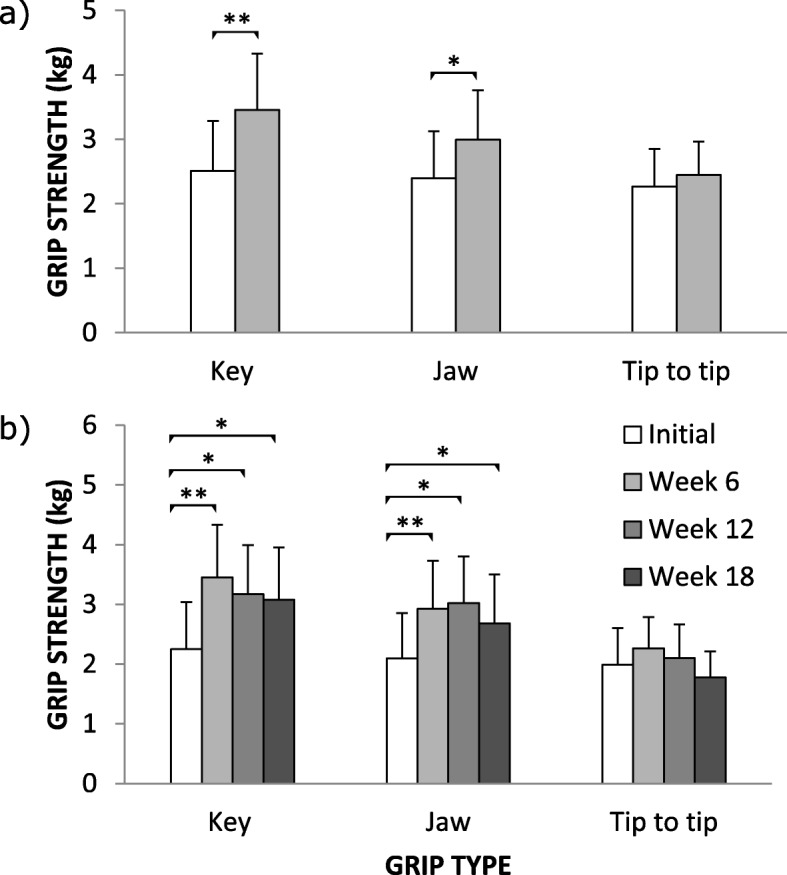
Results of dynamometry using pinchmeter. Strength test using pinchmeter for all participants who **a** completed up to two assessment sessions and **b** completed all study sessions. In **a** Key, *n*=12; Jaw, *n*=11 and Tip to tip, *n*=11. In **b***n*=9. In **a** *, *p* <0.05; **, *p* <0.01. In **b** *, *p* <0.016; *, *p* <0.003

The result of the strength test is shown in Fig. 3. The means were significantly greater in Week 6 compared to the Initial for only the key and jaw grip types. The mean increased from 2.5 ±0.8 kg to 3.5 ±0.9 kg (*p* = 0.0036, *n*=12) for key grip and 2.4 ±0.7 kg to 2.9 ±0.8 kg (*p*= 0.023, *n*=11) for jaw grip but remained similar (2.3 ±0.6 kg to 2.4 ±0.5 kg ; *p*>0.1, *n*=11) for the tip to tip grip.

The repeated measures analysis of the key grip strength results revealed a main effect of assessment session, F(3,24)= 8.6075, *p*= 0.0004686 (*n*=9). Post hoc analysis using Bonferoni correction for multiple comparison revealed a significant increase in pinch strength from the Initial assessment session (2.2 ±0.8 kg) to Week 6 (3.5 ±0.9 kg, *p* = 0.0009893), Week 12 (3.2 ±0.8 kg, *p* = 0.01629), and Week 18 (3.1 ±0.9 kg, *p* = 0.008444).

Similarly the repeated measures analysis for the jaw grip strength results revealed a main effect of assessment session, F(3,24)= 7.7558, *p*= 0.0008620 (*n*=9). Post hoc analysis using Bonferoni correction for multiple comparison revealed a significant increase in pinch strength from the Initial assessment session (2.1 ±0.8 kg) to Week 6 (2.9 ±0.8 kg, *p* = 0.001438), Week 12 (3.0 ±0.8 kg, *p* = 0.01017), and Week 18 (2.7 ±0.8 kg, *p* = 0.008062).

In contrast, the repeated measures analysis of the tip to tip grip strength results did not reveal a main effect of assessment session, F(3,24)=0.89017, *p*= 0.4604 (*n*=9).

### Modified ashworth scale

Wilcoxon signed rank test (*n* = 12) showed that there was no significant difference between the MAS score for the Initial and Week 6 assessments for each of the shoulder (*p* = 1.000), elbow (*p* = 0.533), wrist (*p* = 1.000), fingers (*p* = 1.000) and thumb (*p* = 0.109).

**Fig. 4:**
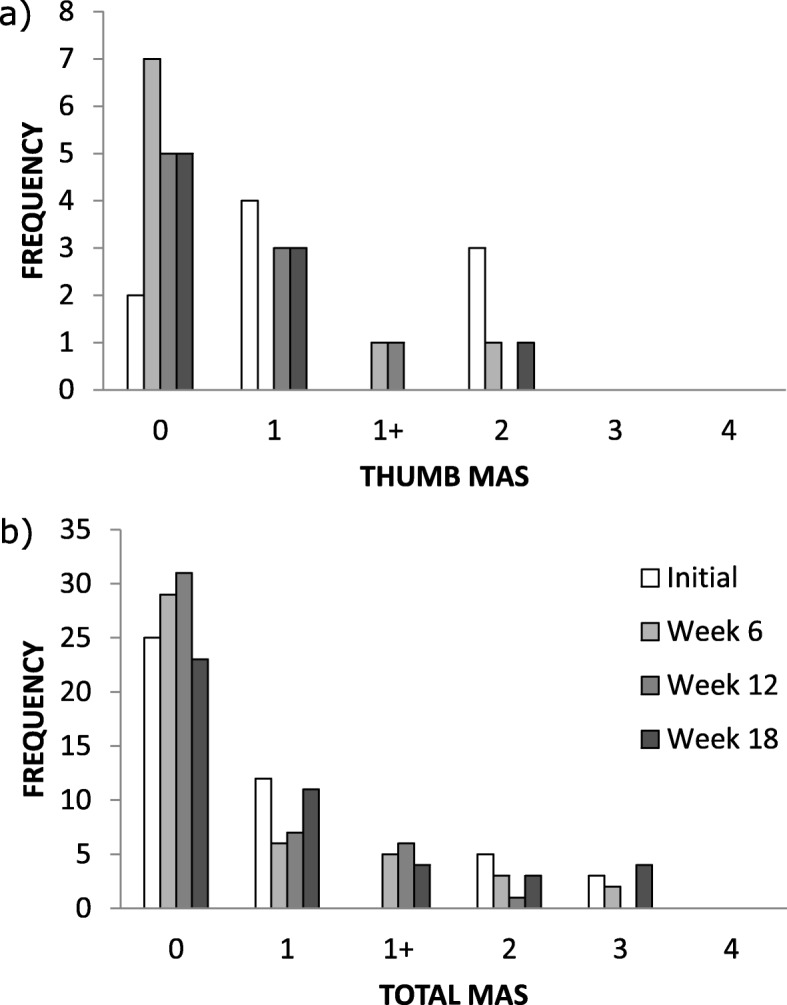
Results of modified Ashworth scale. Frequency of each point on the Modified Ashworth scale summed across all participants with complete data and for **a** all assessed upper limb movements, **b** the thumb alone

Figure 4 shows an aggregate frequency plot of MAS scores for the total and for the thumb alone. The figure shows that the participants were assessed to have normal muscle tone (MAS score = 0) more frequently during the Week 6 (frequency of 0 = 29) and Week 12 (frequency of 0 = 31) assessments which correspond to the period of glove use (cf. Initial, frequency of 0 =25; Week 18, frequency of 0= 23). However, the highest MAS score representing abnormal muscle tone registered in this study (MAS score = 3) was more frequent during the Initial (frequency of 3 = 3) and Week 18 (frequency of 3 = 4) assessments which correspond to the period that the glove was not in use (cf. Week 6, frequency of 3 =2; Week 12, frequency of 3= 0). Friedman test on the MAS data over the four assessment sessions showed no significant effect of assessment session for the shoulder *χ*^2^(3) = 6.98, *p* = 0.0725; the elbow *χ*^2^(3) = 3.4528, *p* = 0.3269; the wrist *χ*^2^(3) = 4.71, *p* = 0.194 and for the fingers *χ*^2^(3) = 1.38, *p* = 0.7106. However the test showed significant effect of assessment session for the thumb, *χ*^2^(3) = 8.88, *p* = 0.0309 (*n*=7). Post hoc analysis for the thumb revealed there was a significant difference between the Initial and Week 6 (*p* = 0.0053) and an almost significant change between Initial and Week 12 (*p* = 0.0212). This result suggests there was a significant decrease in the measured muscle tone among the study participant especially within the first six weeks of the study. However this improvement was not sustained because the effect was not significant at the Week 12 assessment and there was no significant difference in muscle tone between Initial and Week 18 (*p* = 0.0896).

### SF-36 and standard gamble health state valuation

No significant change in total SF-36 score was identified between the Initial (3.5, 3.0 - 4.0; median, 25th - 75th percentile) and Week 12 (4.0, 3.5 - 4.0). In addition, no change in the Standard Gamble health state valuation was observed between Initial (0.57, 0.47 - 0.56) and Week 12 (0.56, 0.54 - 0.59).

## Discussion

This study has shown that a home-based activity-based self-administered therapy using an assistive device leads to improvement in hand function in people with chronic SCI.

The TRI-HFT and grip strength test using pinchmeter showed improvement in hand function starting from the first six weeks of the study. The improvements were generally maintained at follow-up, suggesting that the glove induced long-lasting changes on prehension and grip strength, which are important for object manipulation during ADL. For example an improvement in pinch grip strength and the wooden bar component of the TRI-HFT reflect an improvement in palmar grasp strength, essential to perform ADL such as holding a pan, knife, toothbrush, hairbrush and shaver [45]. Although the minimal clinically important difference has yet to be defined for the TRI-HFT, the dramatic change in the wooden bar, instrumented cylinder and credit card components of the TRI-HFT which was maintained at least up to Week 12, would facilitate object manipulation enabling participants to improve in the performance of ADL.

Some of the TRI-HFT components demonstrated either an almost significant improvement in hand function or no improvement at all (e.g the wooden block component). This is likely due to the high initial scores from the participants in this study who have less severe SCI (AIS C and D) (c.f [50] and [51]) prior to the therapy. It is also possible that some components of the TRI-HFT were not sufficiently sensitive to detect all relevant functional changes.

Other measures that did not show marked differences include the ASIA examination and SF-36. It is important to note however that ASIA examination was used in this study mainly to determine the exact classifications of the participants’ injury and to determin suitaility for inclusion. The ASIA, ISNCSCI sensorimotor scores only significantly improved for the pinprick of the ipsilateral C5-T1 dermatomes and not for the light touch, or UEMS which would directly reflect motor improvement. The subjective nature of ASIA and the SF-36 may have affected their scores, considering other improvement recorded in this study. Testimonies from participants and video recordings support the improvement in function recorded with the more objective measures. Video recordings during the tests (Additional files 1, 2 and 3) suggest a dramatic improvement in participants’ object manipulation capability, including speed and quality of movement (or smoothness of hand trajectory). For example, as shown in Additional file 1, a participant who struggled to manipulate a can to simulate drinking during the Initial assessment was able to smoothly perform this task during the Week 6 session.

Furthermore, participants in this study who have chronic SCI (14 -192 months) may also have learnt compensatory strategies to perform ADL using adapted passive movement strategies, e.g tenodesis grasp and release. It is possible that the SEM Glove impeded the use of the learned compensatory movement strategies in participants [50], due to the design of the wrist strap, while encouraging active movements as much as possible. While the SEM Glove may facilitate increased hand strength (or even range of movement), it is unclear whether adaptive compensatory movements are also affected eg. the use of tenodesis grip. Given that the TRI-HFT penalises the use of passive strategies like tenodesis, improvement in strength alone in participants using such strategies may not be detected with this test but may be instead observed in the speed and smoothness of movement trajectories as shown in the videos.

Assessment of hand muscle spasticity to detect hypertonia using the MAS [46] revealed a significant improvement in thumb muscle tone especially at the Week 6 assessment session. Unlike the robust functional improvement observed in this study, the change in MAS score was present when all study sessions were analysed together but not when only the Initial and Week 6 sessions which had a larger sample size were compared (participants CP010, CP012, CP013 have missing or inadequate data for Week 12 and 18). It is possible that the effect on muscle hypertonia requires a longer time to be evident. Muscle hypertonia includes some changes in passive muscle tone which may require a minimum length of rehabilitation to overcome these structural changes in muscle properties [52]. Also it is surprising, given that the device used in this study was designed for gross grasping, that only the thumb demonstrated improved tone scores during the therapy. Similar improvements in general hand muscle spasticity have also been observed in a home-based robotic rehabilitation therapy for stroke assessed using MAS [19].

Most of the participants believed that they benefited from using the SEM Glove and stated that they used the device for various activities including eating, holding a handrail while climbing stairs, grooming, driving and also for other bimanual tasks. They reported improved function such as greater hand grip strength and dexterity at the follow-up assessment session. QUEST analysis highlighted that effectiveness, comfort, and ‘ease to use’ were the most important satisfaction items, and that the participants were more or less satisfied with the device. The participant’s overall evaluation of ‘quite satisfied’ on the QUEST scale suggested that they accepted the device. This is in agreement with results of a recent study among elderly people, where it was reported that the participants assessed the SEM Glove system as usable during ADL [53]. Some of the participants in the present study expressed interest and wanted to keep the device at the end of the study. Although they were impressed, they also made some recommendations which included adding more sensors to the device to provide control for all fingers and an addition of a hand extension mechanism. Most of the participants in this study noted that they had difficulty extending their hands. They therefore particularly stressed the need for the device to facilitate hand extension. From the experience of running the current study we believe that the glove under study and similar devices are likely to appeal to more people with impaired hand function (due to SCI at least) if they support both hand extension and flexion.

People with severe SCI may be able to use the glove if they have some residual movement sufficient to activate the device. This study indicates that the glove should be initially tested by the participant to better assess the potential usability of the device. A future study should also determine the ability of the participant to activate and deactivate the glove, as an inclusion criterion. This will help to make sure that the device is investigated with people who find it suitable for performing ADL. In this study, a few participants, especially those who withdrew from the study, did not believe that the device was suitable for them when performing ADL. Some of these participants found that the device was obstructing instead of helping during ADL. These participants have possibly adapted or have improved naturally significantly since their injuries. It seemed that the participants had high expectations of the capability of the glove and one talked about being disappointed because of the expectation that the device could be used for more activities (e.g. wet activities).

Adherence is one of the factors that affect effectiveness of a therapy. For a self-administered therapy in a home setting, adherence is affected by familiarity with the involved technology and availability due to competing commitments of users [54, 55]. Indeed a study showed that adolescents with SCI were unable to dedicate time to home-based functional electrical stimulation for standing and had issues with some aspects of the technology considering the therapy as a separate occasional activity [55]. A home-based therapy programme implemented using a user friendly technology which incorporates normal daily activities and does not involve excessive time commitment has the potential for high user adherence. This is supported by a result from a recent study using the device used here to improve hand function among elderly people [53]. The interesting result from that study is that participants who used the device for ADL had a significantly higher adherence than those who had to use the device to play a computer game. Although the computer game may be interesting and motivational, participants whose tasks were simply incorporated into ADL had a better adherence. The advantage of the device used in this study over many rehabilitation and asisstive devices for the upper limb (see [26, 27] and [28] for reviews), e.g the system used in [20] and [21], is its simplicity which means that no training is required (cf. [22]) and the possibility of incorporating its use into normal daily activity thereby reducing users’ time commitment.

Although adherence in this study was below the trial design recommendation, the results are encouraging for a home-based self-administered therapy. On average participants reported that they used the device for 20 h/week during the first six weeks and 15 h/week thereafter which is higher than 3.7 h/week in a recent study using the same device used here [53]. This usage amount which is possibly an overestimation is also much higher than a therapy session duration in clinics which is usually an hour per day during weekdays; although apart from associated costs and availability, patients are likely going to benefit more from therapist guided sessions

The changes in hand function among the participants include improvement in pinch grip which is a fine motor function. This may seem surprising since the device’s overall function mainly supports gross grasping. However the gross grasping is achieved by pulling on the thumb and individual fingers under the control of the device. Improvement in the gross grip will also likely benefit more intricate grip types since the same digits, which may have been strengthened, are used to perform both grips. In essence, hand function is mediated by synergistic sets of muscles [56], where improvement in one synergy is likely to benefit another since muscles are shared between synergies. Furthermore, people often adapt following impairment of the hand and may adapt to use the device for tasks requiring intricate hand movements. One of the participants who discontinued from the study was able to write (a task requiring intricate hand movement) by adapting the device during the Initial assessment session. Thus, although the device is designed for gross grasping, it can also lead to improvement in fine motor function.

Care should be made when interpreting some of the results of this study. Firstly, the number of participants was low and few individuals dropped out of the study before the Week 12 assessment. As such data presented for the Week 12 of the intervention period may not be directly compared to the improvement in hand function observed during the Week 6, or with the follow-up period. Secondly, in this pilot study no control group was included to compare the effect of the glove therapy.

However, the consistent therapeutic results, even under rigorous analysis which included Bonferroni correction for multiple comparison, indicated that functional recovery could be improved and maintained with the SEM Glove at home during the chronic stage of SCI. Application of SEM Glove therapy during the subacute stage of injury may provide further evidence of recovery of function much earlier after SCI, when spontaneous but limited motor recovery is normally present.

These results did not indicate the mechanism that led to the improvement in function. The improvements may be due to changes at the level of the muscle and/or, given that the glove encourages active participation, at the spinal and supraspinal levels due to neuroplasticity. This and several other issues such as the minimal residual hand muscle function required to use the device, the minimal therapy time required to detect a functional effect and how long the effect is maintained, need to be clarified. For example, it may take less than six weeks to observe a therapeutic effect as demonstrated in a recent study among elderly people where improvements were observed after four weeks [53] of intervention. A future controlled multicentre trial with a larger sample size will help to clarify these and validate this home-based rehabilitation as an affordable and accessible programme for upper limb therapy for people with high level SCI.

## Conclusion

This uncontrolled study demonstrates that an intensive home-based self-administered therapy for 12 weeks in people with chronic SCI leads to a significant improvement in unassisted hand and pinch function which is retained at 6 weeks follow-up. This suggests that similar activity-based rehabilitation programmes could provide an affordable and accessible rehabilitation strategy for discharged patients who do not have regular access to therapy. This result should inspire more home-based studies to investigate the feasibility of delivering accessible self-administered upper limb therapies to improve ADL performance for people with tetraplegia living in the community. Future studies should characterise the neuromuscular mechanism of action and the minimal rehabilitation programme necessary with the assistive device to improve ADL tasks following chronic cervical SCI.

## Supplementary information

**Additional file 2** A video of a participant completing a task involving manipulation of a book from Toronto Rehabilitation Institute hand function test. The participant was instructed to pick up the book at the spin and manipulate it; turning the palm up and down while still holding it. The video shows the performance on this task for the participant during the baseline assessment (Initial), after six weeks of using the device (Week 6), after 12 weeks of using the device (Week 12) and during the follow up assessment session (Week 18). The task was more difficult and was performed less smoothly at initial assessment session compared with the other assessment sessions.

**Additional file 3** A video of a participant completing a task involving manipulation of a card from Toronto Rehabilitation Institute hand function test. The participant was instructed to pick up the card and manipulate it; turning the palm up and down while still holding it. An example of this task in activities of daily living is the use of a bank/credit card. The video shows the performance on this task for the participant during the baseline assessment (Initial), after six weeks of using the device (Week 6), after 12 weeks of using the device (Week 12) and during the follow up assessment session (Week 18). The task was more difficult and was performed less smoothly at initial assessment session compared with the other assessment sessions.

## Data Availability

Data resulting from this study is stored in accordance with the Buckinghamshire Healthcare NHS Trust’s policy. Anonymised datasets generated and/or analysed during the current study are available from the corresponding author on reasonable request.

## Abbreviations

ADL: Activities of daily living
AIS: ASIA impairment scale
ASIA: American spinal injury association
ISNCSCI: International standards for neurological classification of spinal cord injury
LT: Light touch
MAS: Modified Ashworth scale
NSIC: National spinal injuries centre
PP: Pinprick
QUEST: Quebec user evaluation of satisfaction with assistive technology
SCI: Spinal cord injury
SEM: Soft extra muscle
SF-36: Short form 36
TRI-HFT: Toronto rehabilitation institute hand function test
UEMS: Upper extremity motor score

## References

[1] Lo C, Tran Y, Anderson K, Craig A, Middleton J (2016). Functional priorities in persons with spinal cord injury: using discrete choice experiments to determine preferences. J Neurotrauma.

[2] Anderson KD (2004). Targeting recovery: priorities of the spinal cord-injured population. J Neurotrauma.

[3] Heo P, Gu GM, Lee S-j, Rhee K, Kim J (2012). Current hand exoskeleton technologies for rehabilitation and assistive engineering. Int J Precis Eng Manuf.

[4] Wege A, Hommel G. Development and control of a hand exoskeleton for rehabilitation of hand injuries. In: Intelligent Robots and Systems, 2005.(IROS 2005). 2005 IEEE/RSJ International Conference On. IEEE: 2005. p. 3046–51. 10.1109/iros.2005.1545506.

[5] Wege A, Zimmermann A. Electromyography sensor based control for a hand exoskeleton. In: Robotics and Biomimetics, 2007. ROBIO 2007. IEEE International Conference On. IEEE: 2007. p. 1470–5. 10.1109/robio.2007.4522381.

[6] Hasegawa Y, Mikami Y, Watanabe K, Sankai Y. Five-fingered assistive hand with mechanical compliance of human finger. In: Robotics and Automation, 2008. ICRA 2008. IEEE International Conference On. IEEE: 2008. p. 718–24. 10.1109/robot.2008.4543290.

[7] Tadano K, Akai M, Kadota K, Kawashima K. Development of grip amplified glove using bi-articular mechanism with pneumatic artificial rubber muscle. In: Robotics and Automation (ICRA), 2010 IEEE International Conference On. IEEE: 2010. p. 2363–8. 10.1109/robot.2010.5509393.

[8] Tong KY, Ho SK, Pang PMK, Hu XL, Tam WK, Fung KL, Wei XJ, Chen PN, Chen M. An intention driven hand functions task training robotic system. In: Engineering in Medicine and Biology Society (EMBC), 2010 Annual International Conference of the IEEE. IEEE: 2010. p. 3406–9. 10.1109/iembs.2010.5627930. 10.1109/IEMBS.2010.5627930 21097247

[9] Li J, Zheng R, Zhang Y, Yao J. iHandRehab: An interactive hand exoskeleton for active and passive rehabilitation. In: Rehabilitation Robotics (ICORR), 2011 IEEE International Conference On. IEEE: 2011. p. 1–6. 10.1109/icorr.2011.5975387. 10.1109/ICORR.2011.5975387 22275591

[10] Hasegawa Y, Tokita J, Kamibayashi K, Sankai Y. Evaluation of fingertip force accuracy in different support conditions of exoskeleton. In: Robotics and Automation (ICRA), 2011 IEEE International Conference On. IEEE: 2011. p. 680–5. 10.1109/icra.2011.5980512.

[11] Polygerinos P, Wang Z, Galloway KC, Wood RJ, Walsh CJ (2015). Soft robotic glove for combined assistance and at-home rehabilitation. Robot Auton Syst.

[12] Kadowaki Y, Noritsugu T, Takaiwa M, Sasaki D, Kato M (2011). Development of soft power-assist glove and control based on human intent. J Robot Mechatron.

[13] Kang BB, Choi H, Lee H, Cho K-J (2019). Exo-glove poly ii: A polymer-based soft wearable robot for the hand with a tendon-driven actuation system. Soft Robot.

[14] Heung KH, Tong RK, Lau AT, Li Z (2019). Robotic glove with soft-elastic composite actuators for assisting activities of daily living. Soft Robot.

[15] Xiloyannis M, Cappello L, Khanh DB, Yen S-C, Masia L. Modelling and design of a synergy-based actuator for a tendon-driven soft robotic glove. In: 2016 6th IEEE International Conference on Biomedical Robotics and Biomechatronics (BioRob). IEEE: 2016. p. 1213–9. 10.1109/biorob.2016.7523796.

[16] Nycz CJ, Delph MA, Fischer GS. Modeling and design of a tendon actuated soft robotic exoskeleton for hemiparetic upper limb rehabilitation. In: 2015 37th Annual International Conference of the IEEE Engineering in Medicine and Biology Society (EMBC). IEEE: 2015. p. 3889–92. 10.1109/embc.2015.7319243. 10.1109/EMBC.2015.7319243 26737143

[17] Da-Silva RH, Moore SA, Price CI. Self-directed therapy programmes for arm rehabilitation after stroke: a systematic review. Clin Rehabil. 2018:0269215518775170. 10.1177/0269215518775170. 10.1177/0269215518775170 29756513

[18] Holden MK, Dyar TA, Schwamm L, Bizzi E (2005). Virtual-environment-based telerehabilitation in patients with stroke. Presence Teleoperators Virtual Environ.

[19] Sivan M, Gallagher J, Makower S, Keeling D, Bhakta B, O’Connor RJ, Levesley M (2014). Home-based computer assisted arm rehabilitation (hcaar) robotic device for upper limb exercise after stroke: results of a feasibility study in home setting. J Neuroengineering Rehabil.

[20] Nijenhuis SM, Prange GB, Amirabdollahian F, Sale P, Infarinato F, Nasr N, Mountain G, Hermens HJ, Stienen AH, Buurke JH (2015). Feasibility study into self-administered training at home using an arm and hand device with motivational gaming environment in chronic stroke. J Neuroengineering Rehabil.

[21] Cordo P, Lutsep H, Cordo L, Wright WG, Cacciatore T, Skoss R (2009). Assisted movement with enhanced sensation (ames): coupling motor and sensory to remediate motor deficits in chronic stroke patients. Neurorehabil Neural Repair.

[22] Brown EVD, McCoy SW, Fechko AS, Price R, Gilbertson T, Moritz CT (2014). Preliminary investigation of an electromyography-controlled video game as a home program for persons in the chronic phase of stroke recovery. Arch Phys Med Rehabil.

[23] Coupar F, Pollock A, Legg LA, Sackley C, van Vliet P. Home-based therapy programmes for upper limb functional recovery following stroke. Cochrane Database of Syst Rev. 2012; 5. 10.1002/14651858.cd006755.pub2. 10.1002/14651858.CD006755.pub2 PMC6464926 22592715

[24] Turton A, Fraser C (1990). The use of home therapy programmes for improving recovery of the upper limb following stroke. Br J Occup Ther.

[25] Zhang H, Austin H, Buchanan S, Herman R, Koeneman J, He J. Feasibility studies of robot-assisted stroke rehabilitation at clinic and home settings using rupert. In: Rehabilitation Robotics (ICORR), 2011 IEEE International Conference On. IEEE: 2011. p. 1–6. 10.1109/icorr.2011.5975440. 10.1109/ICORR.2011.5975440 22275640

[26] Chu C-Y, Patterson RM (2018). Soft robotic devices for hand rehabilitation and assistance: a narrative review. J Neuroengineering Rehabil.

[27] Maciejasz P, Eschweiler J, Gerlach-Hahn K, Jansen-Troy A, Leonhardt S (2014). A survey on robotic devices for upper limb rehabilitation. J Neuroengineering Rehabil.

[28] Riener R, Nef T, Colombo G (2005). Robot-aided neurorehabilitation of the upper extremities. Med Biol Eng Comput.

[29] Zondervan DK, Friedman N, Chang E, Zhao X, Augsburger R, Reinkensmeyer DJ, Cramer SC. Home-based hand rehabilitation after chronic stroke: Randomized, controlled single-blind trial comparing the musicglove with a conventional exercise program. J Rehabil Res Dev. 2016; 53(4). 10.1682/jrrd.2015.04.0057. 10.1682/JRRD.2015.04.0057 27532880

[30] Bernocchi P, Mulè C, Vanoglio F, Taveggia G, Luisa A, Scalvini S (2018). Home-based hand rehabilitation with a robotic glove in hemiplegic patients after stroke: a pilot feasibility study. Top Stroke Rehabil.

[31] Prange-Lasonder GB, Radder B, Kottink AI, Melendez-Calderon A, Buurke JH, Rietman JS. Applying a soft-robotic glove as assistive device and training tool with games to support hand function after stroke: Preliminary results on feasibility and potential clinical impact. In: Rehabilitation Robotics (ICORR), 2017 International Conference On. IEEE: 2017. p. 1401–6. 10.1109/icorr.2017.8009444. 10.1109/ICORR.2017.8009444 28814016

[32] Hara Y, Ogawa S, Tsujiuchi K, Muraoka Y (2008). A home-based rehabilitation program for the hemiplegic upper extremity by power-assisted functional electrical stimulation. Disabil Rehabil.

[33] Sullivan J, Girardi M, Hensley M, Rohaus J, Schewe C, Whittey C, Hansen P, Muir K (2015). Improving arm function in chronic stroke: a pilot study of sensory amplitude electrical stimulation via glove electrode during task-specific training. Top Stroke Rehabil.

[34] Zondervan DK, Augsburger R, Bodenhoefer B, Friedman N, Reinkensmeyer DJ, Cramer SC (2015). Machine-based, self-guided home therapy for individuals with severe arm impairment after stroke: a randomized controlled trial. Neurorehabil Neural Repair.

[35] Kowalczewski J, Chong SL, Galea M, Prochazka A (2011). In-home tele-rehabilitation improves tetraplegic hand function. Neurorehabil Neural Repair.

[36] Nilsson M, Ingvast J, Wikander J, von Holst H. The Soft Extra Muscle system for improving the grasping capability in neurological rehabilitation. In: Biomedical Engineering and Sciences (IECBES), 2012 IEEE EMBS Conference On. IEEE: 2012. p. 412–417. 10.1109/iecbes.2012.6498090.

[37] Radder B, Prange-Lasonder GB, Kottink AI, Gaasbeek L, Holmberg J, Meyer T, Melendez-Calderon A, Ingvast J, Buurke JH, Rietman JS (2016). A wearable soft-robotic glove enables hand support in adl and rehabilitation: A feasibility study on the assistive functionality. J Rehabil Assist Technol Eng.

[38] Rushton DN (2003). Functional Electrical Stimulation and Rehabilitation—an Hypothesis. Med Eng Phys.

[39] Osuagwu BCa, Wallace L, Fraser M, Vuckovic A. Brain-Computer Interface and Functional Electrical Stimulation for Neurorehabilitation of Hand in Sub-acute Tetraplegic Patients - Functional and Neurological Outcomes. In: Proceedings of the 3rd International Congress on Neurotechnology, Electronics and Informatics: 2015. p. 15–23. http://www.scitepress.org/DigitalLibrary/Link.aspx?10.5220/0005650500150023.

[40] Lotze M, Braun C, Birbaumer N, Anders S, Cohen LG (2003). Motor learning elicited by voluntary drive. Brain.

[41] Trincado-Alonso F, López-Larraz E, Resquín F, Ardanza A, Pérez-Nombela S, Pons JL, Montesano L, Gil-Agudo Á. A pilot study of brain-triggered electrical stimulation with visual feedback in patients with incomplete spinal cord injury. J Med Biol Eng:1–14. 10.1007/s40846-017-0343-0.

[42] Osuagwu BC, Wallace L, Fraser M, Vuckovic A (2016). Rehabilitation of hand in subacute tetraplegic patients based on brain computer interface and functional electrical stimulation: a randomised pilot study. J Neural Eng.

[43] Hebb DO (1949). The Organization of Behavior : a Neuropsychological Theory.

[44] Demers L, Weiss-Lambrou R, Ska B (2000). Item analysis of the quebec user evaluation of satisfaction with assistive technology (quest). Assist Technol.

[45] Kapadia N, Zivanovic V, Verrier M, Popovic M (2012). Toronto rehabilitation institute–hand function test: Assessment of gross motor function in individuals with spinal cord injury. Top Spinal Cord Inj Rehabil.

[46] Bohannon RW, Smith MB (1987). Interrater reliability of a modified Ashworth scale of muscle spasticity. Phys Ther.

[47] Wang L, Zhang Z, McArdle JJ, Salthouse TA (2008). Investigating ceiling effects in longitudinal data analysis. Multivar Behav Res.

[48] Forchheimer M, McAweeney M, Tate DG (2004). Use of the sf-36 among persons with spinal cord injury. Am J Phys Med Rehabil.

[49] Green C, Brazier J, Deverill M (2000). Valuing health-related quality of life. Pharmacoeconomics.

[50] Cappello L, Meyer JT, Galloway KC, Peisner JD, Granberry R, Wagner DA, Engelhardt S, Paganoni S, Walsh CJ (2018). Assisting hand function after spinal cord injury with a fabric-based soft robotic glove. J Neuroengineering Rehabil.

[51] Soekadar S, Witkowski M, Gómez C, Opisso E, Medina J, Cortese M, Cempini M, Carrozza M, Cohen L, Birbaumer N (2016). Hybrid eeg/eog-based brain/neural hand exoskeleton restores fully independent daily living activities after quadriplegia. Sci Robot.

[52] Mayer NH (2018). New treatment approaches on the horizon for spastic hemiparesis. PM&R.

[53] Radder B, Prange-Lasonder GB, Kottink AI, Holmberg J, Sletta K, van Dijk M, Meyer T, Melendez-Calderon A, Buurke JH, Rietman JS (2019). Home rehabilitation supported by a wearable soft-robotic device for improving hand function in older adults: A pilot randomized controlled trial. PloS ONE.

[54] Standen PJ, Threapleton K, Connell L, Richardson A, Brown DJ, Battersby S, Sutton CJ, Platts F (2015). Patients’ use of a home-based virtual reality system to provide rehabilitation of the upper limb following stroke. Phys Ther.

[55] Moynahan M, Mullin C, Cohn J, Burns CA, Halden EE, Triolo RJ, Betz RR (1996). Home use of a functional electrical stimulation system for standing and mobility in adolescents with spinal cord injury. Arch Phys Med Rehabil.

[56] Scano A, Chiavenna A, Molinari Tosatti L, Müller H, Atzori M (2018). Muscle synergy analysis of a hand-grasp dataset: a limited subset of motor modules may underlie a large variety of grasps. Front Neurorobotics.

